# Giant Cell Arteritis after COVID-19 Vaccination with Long-Term Follow-Up: A Case Report and Review of the Literature

**DOI:** 10.3390/medicina59122127

**Published:** 2023-12-06

**Authors:** Kiyomi Yoshimoto, Saori Kaneda, Moe Asada, Hiroyuki Taguchi, Hiromasa Kawashima, Ryo Yoneima, Hidetoshi Matsuoka, Emiko Tsushima, Shiro Ono, Masaki Matsubara, Noritaka Yada, Kenji Nishio

**Affiliations:** 1 Department of General Medicine, Nara Medical University Hospital, Kashihara 634-8522, Nara, Japan; sa0ri.uetani@gmail.com (S.K.); asamo12@naramed-u.ac.jp (M.A.); taguchi4161@gmail.com (H.T.); kawashima.h@naramed-u.ac.jp (H.K.); y_ryo1122@yahoo.co.jp (R.Y.); hmatsu1227@naramed-u.ac.jp (H.M.); emiemi.roku@gmail.com (E.T.); shiroono0207@gmail.com (S.O.); m.matsubara@naramed-u.ac.jp (M.M.); n-yada@naramed-u.ac.jp (N.Y.); knishio@naramed-u.ac.jp (K.N.); 2 Department of General Medicine, Uda City Hospital, Uda 633-0298, Nara, Japan

**Keywords:** GCA, COVID-19 vaccine, SARS-CoV-2, LV-GCA, tocilizumab

## Abstract

Giant cell arteritis (GCA) is a chronic vasculitis that primarily affects the elderly, and can cause visual impairment, requiring prompt diagnosis and treatment. The global impact of the coronavirus disease 2019 (COVID-19) pandemic has been substantial. Although vaccination programs have been a key defense strategy, concerns have arisen regarding post-vaccination immune-mediated disorders and related risks. We present a case of GCA after COVID-19 vaccination with 2 years of follow-up. A 69-year-old woman experienced fever, headaches, and local muscle pain two days after receiving the COVID-19 vaccine. Elevated inflammatory markers were observed, and positron emission tomography (PET) revealed abnormal uptake in the major arteries, including the aorta and subclavian and iliac arteries. Temporal artery biopsy confirmed the diagnosis of GCA. Treatment consisted of pulse therapy with methylprednisolone, followed by prednisolone (PSL) and tocilizumab. Immediately after the initiation of treatment, the fever and headaches disappeared, and the inflammation markers normalized. The PSL dosage was gradually reduced, and one year later, a PET scan showed that the inflammation had resolved. After two years, the PSL dosage was reduced to 3 mg. Fourteen reported cases of GCA after COVID-19 vaccination was reviewed to reveal a diverse clinical picture and treatment response. The time from onset of symptoms to GCA diagnosis varied from two weeks to four months, highlighting the challenge of early detection. The effectiveness of treatment varied, but was generally effective similarly to that of conventional GCA. This report emphasizes the need for clinical vigilance and encourages further data collection in post-vaccination GCA cases.

## 1. Introduction

Coronavirus disease 2019 (COVID-19) is a fatal viral infection caused by the severe acute respiratory syndrome coronavirus 2 (SARS-CoV-2) virus that has become a global pandemic. The vaccine serves as the primary defense against SARS-CoV-2 infection, and has demonstrated high efficacy in preventing severe cases. Adverse reactions reported after COVID-19 vaccination usually include injection site reactions and mild systemic symptoms, such as chills, fever, arthralgia, myalgia, and headache. Additionally, it has been observed that the vaccine was associated with the onset or exacerbation of immune-mediated diseases [1].

Giant cell arteritis (GCA) is chronic vasculitis characterized by granulomatous inflammation that affects large- and medium-sized arteries [2]. GCA occurs over the age of 50 years, with a peak incidence between 70 and 80 years [3,4]. Clinically, GCA usually presents as an insidious course with general symptoms, including fever, asthenia, or weight loss, along with local symptoms due to arterial inflammation and vascular deficits, such as headache or jaw claudication [3]. GCA can cause acute irreversible visual loss; therefore, prompt diagnosis and treatment initiation are essential. Biopsy specimens containing arteries show necrotizing arteritis characterized by a predominance of mononuclear cell infiltrates or a granulomatous process with multinucleated giant cells [5].

Although the etiology of GCA has not been fully elucidated, environmental factors and infections, particularly respiratory-tract infections, have long been thought to contribute to its pathogenesis [6,7,8]. Some reports have pointed out the potential role of certain viruses, such as cytomegalovirus or varicella zoster, as well as influenza vaccines, as triggers for GCA in predisposed individuals [9,10,11]. Furthermore, GCA that develops after COVID-19 vaccination has been reported [12,13,14,15,16,17,18,19,20,21,22,23], but its characteristics are not clear. Here, we present a case of GCA after COVID-19 vaccination with long-term follow-up and characterize GCA that developed after the COVID-19 vaccine.

## 2. Case Presentation

The patient was a 69-year-old Japanese female with no specific medical history or medications. She received the first doses of an mRNA vaccine against COVID-19 (BNT162b2) in 2021. After two days, she developed swelling and myalgia at the vaccination site, fever (37.2 °C), and headache. Two weeks later, the headache improved, but the fever persisted, and abdominal pain appeared. Blood examination on day 15 showed an elevated C-reactive protein (CRP) level of 238 mg/L and 1-h erythrocyte sedimentation rate (ESR 1 h) of over 140 mm. After 22 days, the patient developed temporary severe abdominal pain, followed by back and lumbar pain. Symptoms of nausea, diarrhea, respiratory symptoms, urinary frequency, pain during urination, residual urine, skin rash, visual field abnormalities, or jaw claudication were not observed.

After 24 days, the patient was referred to our hospital. She exhibited weight loss of 7 kg in 1 month. Physical examination revealed a body temperature of 37.2 °C, blood pressure of 126/62 mmHg, and pulse rate of 110 beats/min. Auscultation confirmed the absence of abnormal lung sounds. Her heartbeat was regular, with no abnormal sounds. Abdominal examination revealed tenderness near the umbilicus and knock pain in the right costovertebral angle (CVA). No cutaneous rashes were observed. Fundus examination did not reveal any sign of ischemic optic neuropathy.

Blood tests revealed anemia (hemoglobin 7.6 g/dL) and an increase in platelet count (58.6·10^4^/μL), along with elevated markers of inflammation (ESR 1 h > 140 mm, CRP 211 mg/L, 50% hemolytic complement activity (CH50) 63 U/mL, complement component 3 (C3) 156 mg/dL, complement component 4 (C4) 37 mg/dL), and mild liver damage (Alanine transaminase (ALT) 40 U/L). Furthermore, mild elevations were observed in ferritin (507 ng/mL), soluble interleukin-2 receptor (712 U/mL), and procalcitonin (0.12 ng/mL). The antinuclear antibody titer was 80-fold, and the IgA, IgG, IgM and IgG4 levels were within the normal range. PR3-ANCA and MPO-ANCA were negative. Polymerase chain reaction (PCR) for SARS-CoV-2 and blood culture tests were negative (Table 1). Anti-double-stranded DNA antibodies, Anti-topoisomerase I (Anti-Scl-70) antibodies, Anti-Sm antibodies, Anti-TIF1-γ autoantibodies, Anti-Mi-2 antibodies, and Anti-RNA polymerase III antibodies were tested and were not detected.

**Figure 1:**
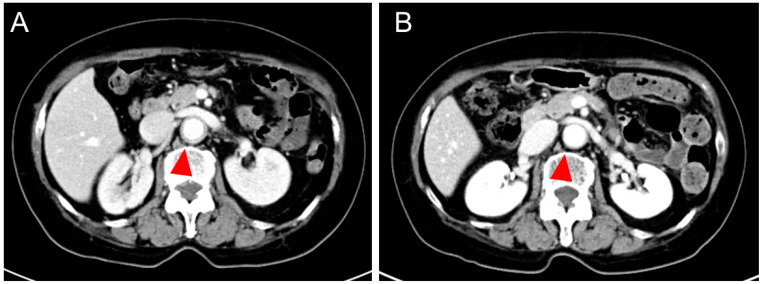
Contrast-enhanced CT at onset of disease (day 24 after vaccination) in 2021 (**A**) showed thickening of the aortic vessel wall (arrows). Contrast-enhanced CT on day 22 of treatment (day 63 after vaccination) (**B**) showed improvement in thickening of the aortic vessel wall (arrows).

**Figure 2:**
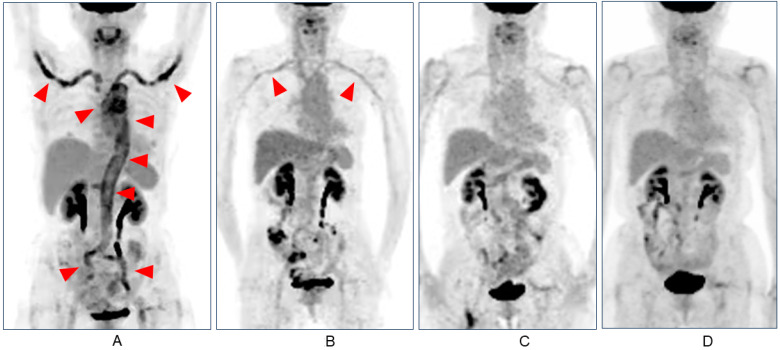
(**A**) PET scan on day 38 after vaccination showed an abnormal uptake in all aortas, bilateral common iliac arteries, and both subclavian arteries (arrows); (**B**) on day 41 of treatment (day 82 after vaccination), there was a slight residual uptake in the subclavian artery (arrows), while uptake in the aorta and iliac arteries significantly reduced; (**C**) PET scan after one year and (**D**) two years, showing that the abnormal uptake has disappeared.

Contrast-enhanced CT on day 24 revealed thickening of the aortic vessel wall (Figure 1A); contrast-enhanced abdominal magnetic resonance imaging (MRI) on day 34 showed an enhancing effect of the vessel wall from the descending aorta to the abdominal aorta. Positron emission tomography (PET) on day 38 showed abnormal uptake in all aortas, the bilateral common iliac arteries, and both subclavian arteries (Figure 2A). MRI of the head revealed no abnormalities.

**Figure 3:**
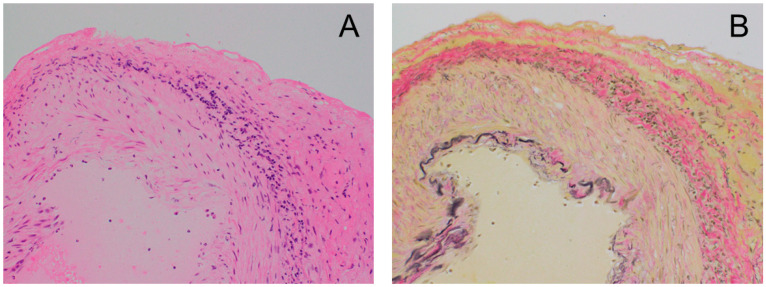
Temporal artery biopsies showed no giant cells, but the adventitia showed mild to moderate infiltration with inflammatory cells, mainly lymphocytes and histiocytes. (**A**) Hematoxylin and eosin staining; (**B**) Verhoeff–Van Gieson elastic staining; magnification: ×100.

On day 40, the patient was admitted to the hospital and a temporal artery biopsy was performed. The biopsy showed no giant cells, but the adventitia showed mild to moderate infiltration with inflammatory cells, mainly lymphocytes and histiocytes (Figure 3). Diagnosis of GCA was performed according to the ACR classification criteria (1990), with at least three of the following five criteria being met: (1) age > 50 years; (2) new-onset local headache; (3) tenderness in the temporal artery or decreased pulse unrelated to atherosclerosis; (4) erythrocyte sedimentation rate > 50 mm/h; and (5) vasculitis with mononuclear cell infiltration, granulomatous inflammation, or multinucleated giant cells in a biopsy specimen of the shallow temporal artery [5]. The present case met criteria (1), (4), and (5), thus GCA was diagnosed.

Treatment was started on day 41 (day 1 of treatment) with pulse therapy of 1000 mg of methylprednisolone for 3 days, followed by the daily prescription of oral dose of 50 mg of prednisolone (PSL). Subcutaneous injection of 162 mg tocilizumab (TCZ) was initiated on day 11 after the start of treatment (day 51 after vaccination). On day 13 of treatment, the fever, headache, and abdominal pain resolved, and the CRP level normalized. After 22 days of treatment, the thickening of the aortic wall improved on contrast-enhanced CT (Figure 1B); 27days later, her anemia and liver damage improved (hemoglobin 10.4 g/dL, ALT 23 U/L) (Table 1). Forty one days later, PET-CT also showed abnormal uptake in aortic wall disappeared, but a slight residual uptake in the subclavian artery (Figure 2B).

The PSL dose was then tapered, and after one year, the inflammatory findings on PET-CT decreased and almost disappeared. (Figure 2C). Two years after the start of treatment, the PSL dose was reduced to 3 mg and PET-CT showed no inflammatory findings (Figure 2D); we continued to taper prednisolone.

## 3. Discussion

We report a case of GCA that developed after COVID-19 vaccination, which had been followed for 2 years. During the SARS-CoV-2 pandemic, there have been several reports of GCA occurring after COVID-19 vaccination. We conducted a search of PubMed, and identified 13 cases of GCA reported in individuals following COVID-19 vaccination [12,13,14,15,16,17,18,19,20,21,22,23] (Table 2). Of the 14 cases, seven males and seven females, four cases of viral vector vaccine and ten cases of mRNA vaccine were observed. The age of onset in these cases ranged from 62 to 87 years (median: 74 years).

In our case, fever and headache appeared 2 days after vaccination, and persisted until the start of treatment. In the reported cases, as in our case, all GCA occurred within a few days after vaccination; however, COVID-19 vaccination can induce acute-onset headache and systemic upset, including fever, fatigue, and myalgia, mimicking the presentation of giant cell arteritis. These symptoms persisted in most cases, but it is not possible to determine when they can be considered symptoms of GCA. These facts delay the diagnosis of post-vaccine GCA. We took 38 days (approximately 5 weeks) to diagnose the patient with GCA. In the 14 reported cases, the time from onset to diagnosis ranged from a minimum of 2 weeks to a maximum of 4 months, and the average time was approximately 6 weeks.

We reviewed whether the diagnosis of GCA after COVID-19 vaccination was delayed or not compared with that of conventional GCA. A recent study analyzing 61 cases with conventional GCA from 2015 to 2017 reported the time from onset to diagnosis to be 4 weeks [24], suggesting that the diagnosis of GCA after COVID-19 vaccination may be delayed. Considering that blindness has occurred in 2 of the 14 post-vaccine GCA cases, early diagnosis should be kept in mind.

This case had a very high CRP level of 211 mg/L, anemia and liver dysfunction, which were considered to be due to GCA, because all of these had improved by day 27 after the start of treatment for GCA. Among the other cases, four out of ten patients in the mRNA vaccine group and one out of four patients in the viral vector group had a high CRP level of 100 mg/L or higher, suggesting that the mRNA group may be more likely to have GCA with a high inflammatory level.

In this case, abnormal uptake in the PET was observed in the aortic arch, thoracic aorta, abdominal aorta, carotid artery, and subclavian and iliac artery. Although GCA has traditionally been considered a disease of the temporal artery, it is now understood to be a systemic disease involving the aorta and any of its major tributaries [25,26,27]. Three primary disease subtypes are recognized: classical or pure cranial GCA (C-GCA), extracranial manifestations in the context of established cranial disease, and isolated extracranial large vessel disease without cranial manifestations. The latter two are designated as large-vessel GCA (LV-GCA) [28]. Our case was classified as an LV-GCA. Among the cases reviewed in this report, 7 of 14 were LV-GCA. Is this a higher percentage than that of conventional GCA? According to the literature, the percentage of LV-GCA depends on the examination method; autopsy studies have shown histological evidence of large-vessel involvement in 80% of cases [29,30]. Systematic screening of patients using radiographic imaging has yielded a variable prevalence of extracranial involvement, depending on the technique employed. In prospective studies of patients with a new diagnosis of GCA, LV-GCA was observed in 29–83% [31,32,33,34,35]. The incidence of LV-GCA in post-vaccine GCA was not different from that in conventional GCA.

In our case, the temporal artery biopsy showed no giant cells, but adventitia was mildly to moderately infiltrated with inflammatory cells, which was considered an early pathological picture of GCA [36]. In their review of six cases of GCA after COVID-19 vaccine, Wakabayashi et al. [23] reported that temporal artery biopsies are less frequently positive (2/6) for pathological findings than in conventional GCA. However, in the present 14 cases, 8 were positive, which was more frequent than their report. Is the biopsy positivity rate for post-vaccine GCA low? Recently, the sensitivity of the temporal artery has been reported to be low, partially because of the timing and length of sampling. In a 2021 review conducted by Dua et al. [37], across six cohort and case–control studies of 856 patients, the pooled sensitivity of temporal artery biopsy in patients with suspected GCA was 61% (95% CI 38–79%), and the pooled specificity was 98% (95% CI 95–99%). Thus, it is difficult to rule out GCA even when temporal artery biopsy is negative. In fact, among 14 cases of post-vaccine GCA, six of seven cases in the C-GCA group had positive biopsies, but only two of seven cases in the LV-GCA group were positive, so it is necessary to consider the possibility of GCA even if the temporal artery biopsy is negative, especially in the LV-GCA group.

In 2022, the new American College of Rheumatology (ACR) classification criteria for GCA were established [38]. The 1990 ACR criteria [5] mostly focus on cranial features of GCA, and do not perform well in classifying patients with diseases that predominantly affect larger arteries. According to the ACR 2022 GCA classification criteria, the absolute condition is an age of 50 years or older at the time of diagnosis, and a diagnosis is performed if the patient has a score of 6 or more on the following 10 items:

[clinical criteria]
  (1) morning stiffness in the shoulders/neck (+2)
  (2) sudden visual loss (+3)
  (3) jaw or tongue claudication (+2)
  (4) new temporal headache (+2)
  (5) scalp tenderness (+2)
  (6) abnormal examination of the temporal artery (+2)
[test/imaging/biopsy criteria]
  (7) maximum ESR ≥50 mm/hour or maximum CRP ≥ 10 mg/L (+3)
  (8) positive temporal artery biopsy or halo sign on temporal artery ultrasound (+5)
  (9) bilateral axillary involvement (+2)
  (10) PET activity throughout the aorta (+2)

This case met criteria (7), (8), and (10), scoring 10 points, and also met the 2022 classification criteria. Using the 2022 ACR/EULAR classification criteria, we found that patients with the LV-GCA subtype may be identified more easily [39].

Our patient responded relatively well to the initial treatment (methylprednisolone pulse therapy and high-dose prednisolone), but also had extensive vascular inflammation and high CRP levels, and TCZ was used. The steroids dose was then carefully reduced and has been reduced over a period of two years. In 14 cases, although two patients exhibited visual field loss, most responded well to treatment. Most of the treatments consisted of an initial high dose of glucocorticoids, which could be reduced as symptoms improved. Methotrexate was used in one case, and TCZ was used in four cases. A trial published in 2017 [40] demonstrated that TCZ has a significant glucocorticoid-sparing effect on GCA; the 2021 ACR guidelines recommend the use of oral glucocorticoids in combination with TCZ [41]. In one of the 14 post-vaccine GCA cases, severe visual impairment appeared during steroid therapy, but the visual impairment improved with additional use of TCZ.

Our patient had been treated for two years with prednisolone and TCZ. Most of the reviewed cases are still under treatment, and we do not yet know how long they will need to be treated. The treatment of GCA generally requires long-term treatment of two years or more [42], and LV-GCA requires a longer treatment period than C-GCA. In a report by Muratore et al. [43], the median time to reach a daily dose of less than 10 mg of prednisone in LV-GCA was reported to be 1.2 years, compared with 0.9 years in C-GCA; the median time to discontinue corticosteroid therapy was reported to be 4.5 years in the LV-GCA group, significantly longer than the 2.2 years in the C-GCA group. Our patient was a case of LV-GCA, and it is expected to take time to reduce the dose of prednisolone.

Is COVID-19 vaccine a trigger for GCA? The etiology of GCA has not been fully elucidated; however, it has long been thought that environmental factors and infections, particularly respiratory tract infections, contribute to its pathogenesis [6,7,8]. In a study of 1005 cases of GCA, Stamatis et al. [6] found significantly higher rates of respiratory tract infections among patients diagnosed with GCA than among controls. Furthermore, several reports have indicated that influenza vaccines [44] and varicella-zoster vaccines [45] may have a higher incidence of GCA. Considering that GCA is universally recognized as an antigen-driven disease, COVID-19 vaccines, including mRNA and viral vector vaccines, use information from the SARS-CoV-2 spike protein to present antigens to the immune system, triggering a robust and protective immune response, followed by the inflammatory process spreading into the arterial wall [46,47]. Thus, COVID-19 vaccine may induce GCA. The conventional GCA is more common in the elderly, and the average age of the reviewed 14 patients was also elderly: 74 years old. This is presumably because the clearance of antigens that may trigger the onset of GCA is lower in the elderly than in the young, making the elderly more susceptible to GCA [48].

There are questions regarding whether the COVID-19 pandemic and vaccination have influenced the development of GCA. There are several reports on the incidence of GCA, hospitalization rates, and their association with COVID-19 vaccines and COVID-19 [49,50,51,52]. Torres et al. reported a higher incidence of GCA hospitalizations in Spain during the second and fifth waves of COVID-19 and the third COVID-19 vaccination in the elderly. At other times, there were no higher incidences than those at pre-pandemic levels [49]. In Italy, to date, an increase in the incidence of GCA has not been reported, with the rate of newly diagnosed patients being stable over the last 2 years of the pandemic [50,51,52,53].

This report had several important limitations. First, as a single case report, the findings may lack broad applicability, and the conclusions drawn may not be generalizable to other populations. Second, while the report suggests an association between COVID-19 vaccination and GCA onset, it does not establish causation, leaving room for uncertainty and the potential influence of other factors, such as pre-existing conditions. Third, the review’s reliance on 14 reported cases may not provide sufficient data for robust conclusions regarding post-vaccination GCA, and a larger, more diverse sample size is needed. In addition, the literature review may not encompass all relevant cases, potentially limiting the comprehensiveness of the conclusions. Addressing these limitations and conducting further research with larger sample sizes, prospective designs, and extended follow-up periods is crucial for a more comprehensive understanding of the relationship between COVID-19 vaccination and GCA.

## 4. Conclusions

GCA is associated with the risk of disturbance of vision and blindness; therefore, early diagnosis and treatment are important. GCA may develop even after receiving the COVID-19 vaccine, and diagnosis may be delayed as symptoms similar to GCA, such as fever, headache, and muscle pain, may occur after vaccination. Thus, it is important to keep GCA in mind when symptoms of adverse reactions of COVID-19 vaccine persist.

## Figures and Tables

**Table 1:** Blood test at the time of arrival (25 days after vaccination) and 27 days after treatment (68 days after vaccination).

Test Name	At the Time of Arrival	27 Days after Treatment	Reference Range
White blood cells (μL)	8300	8200	3300–8600
Red blood cells (10^4^/μL)	254	336	386–492
Hemoglobin (g/dL)	7.6	10.4	11.6–14.8
Hematocrit (%)	22.9	31.8	35.1–44.4
Platelets (10^4^/μL)	58.6	17.1	15.8–34.8
ESR 1 h (mm)	>140	5	3–5
D-dimer (μg/mL)	1.7	1.4	0.0–1.0
C-reactive protein (mg/L)	211	0.1	0.0–1.4
Aspartate aminotransferase (U/L)	30	16	13–30
Alanine transaminase (U/L)	40	23	7–23
Lactate dehydrogenase (U/L)	137	200	124–222
Alkaline phosphatase (U/L)	106	76	106–322
Gamma glutamyl transpeptidase (U/L)	63	26	9–32
Blood urea nitrogen (mg/dL)	11	18	8–20
Creatinine (mg/dL)	0.7	0.76	0.46–0.79
Total bilirubin (mg/dL)	0.6	0.9	0.4–1.5
Procalcitonin (ng/mL)	0.12	-	<0.05
50% hemolytic complement activity (CH50) (U/mL)	63	-	30–46
Complement component 3 (C3) (mg/dL)	156	-	73–138
Complement component 4 (C4) (mg/dL)	37	-	11–31
IgA (mg/dL)	275	-	93–393
IgG (mg/dL)	1697	-	861–1747
IgM (mg/dL)	55	-	50–269
IgG4 (mg/dL)	91		11–121
PR3-ANCA (U/mL)	<1.0	-	<1.0
MPO-ANCA (U/mL)	<1.0	-	<1.0
Antinuclear Antibodies	1:80	-	<40
Ferritin (ng/mL)	507	370 *	<55
Soluble interleukin-2 receptor (U/mL)	712	--	157–474
Blood culture	Negative	-	Negative
Polymerase chain reaction (PCR) for SARS-CoV-2	Negative	-	Negative

* 8 days after treatment (52 days after vaccination), ESR, erythrocyte sedimentation rate; PR3-ANCA, proteinase 3–antineutrophil cytoplasmic antibodies; MPO-ANCA, myeloperoxidase–antineutrophil cytoplasmic antibodies; Ig, immunoglobulin.

**Table 2:** Cases of giant cell arteritis after COVID-19 vaccination, including this case.

Reference	Sex, Age	Past Medical History	Symptoms	Type of Vaccine	Number of Vx	Time from Vx to Onset	Time from Onset to Dx	Mode of Dx	Result of Biopsy	LV-GCA or C-GCA	Arteritis Location	CRP (mg/L)	ESR (mm/h)	Treatment	Time to Improvement	Treatment Duration	Outcome
Cadiou et al. [12]	F, 70	PMR	Fatigue	Viral vector	2	10 days	3 weeks	PET	Not described	LV-GCA	A panaortic and supra-aortic vasculitis (PET)	104	-	PSL 40 mg	1 month	More than 11 weeks	Discharge
Sauret et al. [13]	M, 70	Not described	Headache, hyperesthesia of the scalp	Viral vector	1	Few days	Not described	Biopsy	GCA	C-GCA	Temporal artery (PET, No large vessel vasculitis)	14	-	PSL 0.5 mg/kg	Following day	Not described	Discharge
Xia et al. [14]	M, 68	Chronic obstructive pulmonary disease	Right-sided temporal headache, blurred vision, bilateral jaw claudication	Viral vector	2	3–5 days	3 weeks	Biopsy	GCA	C-GCA	Temporal artery (Angiogram normal)	29	4	1 g mPSL pulse, PSL 65 mg, TCZ 162 mg	PSL 65 mg only, blurred vision on opposite side after 3 months, improved after 3 months with TCZ	More than 6 months	Discharge
Sardo et al. [15]	M, 78	Melanoma, HTN Hepatitis B infection,	Headache, fatigue, jaw claudication, scotomas, pharyngalgia, dry cough	Viral vector	2	1 day	1 month	Biopsy	GCA	LV-GCA	Ascending aorta, aortic arch, descending aorta, iliac axes bilaterally and subclavian bilaterally (PET)	84	-	PSL 1 mg/kg TCZ	A few weeks	More than 8 months	Discharge
Greb et al. [16]	M, 79	HTN, hyperlipidemia, atrial fibrillation, hypothyroidism, prostate cancer, rectal cancer	Headache, transient blurry vision, fever, fatigue, myalgias	mRNA	2	2 days	1 month	Biopsy	GCA	C-GCA	Temporal artery	272	97	PSL 60 mg	3 weeks	More than 6 weeks	Discharge
Cadiou et al. [12]	F, 74	Advanced ovarian cancer	Headache, jaw claudication	mRNA	1	7 days	5 weeks	Biopsy	GCA	C-GCA	Temporal artery	190	-	PSL 60 mg	1 week	Not described	Discharge
Gambichler et al. [17]	M, 82	Not described	Headaches, jaw claudication, weight loss, bilateral temporoparietal skin necrosis, vision loss	mRNA	2	10 days	4 months	Biopsy	GCA	C-GCA	Temporal artery	63	-	Not described	Not described	Not described	Discharge (Complete vision loss before treatment)
Mejren et al. [18]	F, 62	Not described	Fatigue, weight loss, night sweat, nausea	mRNA	1, 2	1–2 days	7–8 weeks	PET	Not described	LV-GCA	The vertebral, common carotid, maxillary, axillary, subclavian, internal mammary, common iliac arteries, throughout the aorta (PET)	98	-	PSL 40 mg	2 weeks	Not described	Discharge
Anzola et al. [19]	F, 83	Dyslipidemia, HTN	Disruptive cervical pain, headache, scalp tenderness	mRNA	1	1 day	3 weeks	PET	Normal	LV-GCA	Bilateral vertebral artery (PET)	14	71	Pulse steroids, methotrexate, medium-dose steroids	3 months	More than 6 months	Discharge (Remission weekly methotrexate and low-dose steroids)
Che et al. [20]	F, 87	HTN	Visual loss, scalp tenderness, fever	mRNA	1	1 day	2 weeks	Biopsy	GCA	C-GCA	Temporal artery	8	120	Pulse steroids, PSL 60 mg	4 months	More than 6 months	Discharge (Right eye complete vision loss, left eye blurred vision improved)
Gilio et al. [21]	F, 63	HTN	Fatigue, myalgias, low grade fevers, anorexia, headache, arthralgia, stiffness of upper arms, shoulders and neck	mRNA	1	1 day	1 month	PET	Not described	LV-GCA	Aortic arch, thoracic and abdominal aorta, carotid, subclavian arteries (PET)	74	104	PSL 50 mg	4 weeks	Not described (tapering ongoing)	Discharge (Tapering ongoing)
Ishizuka et al. [22]	M, 74	HTN	Cough, left temporal headache	mRNA	3	1 day	2 months	PET	Not described	LV-GCA	Thoracic aorta, subclavian, axillary, brachial and temporal arteries (PET)	63	79	PSL 30 mg	Not described	Not described	Not described (Symptoms improved)
Wakabayashi et al. [23]	M, 77	Type 2 diabetes mellitus, Basedow’s disease, prostate cancer	Fatigue, headache, nodular swelling and tenderness of the bilateral temporal arteries	mRNA	3	1 day	3 months	Echography	Normal	C-GCA	Temporal artery (Echography)	134	62	1 g mPSL pulse PSL 1 mg/kg TCZ 162 mg	16 days	More than 45 days	Discharge (PSL 30 mg)
Yoshimoto et al. (this case)	F, 69	None	Headache, fever, abdominal pain, body weight loss	mRNA	1	2 days	38 days (5 weeks)	PET	GCA	LV-GCA	Ascending aorta, aortic arch, descending aorta, bilateral subclavian and iliac artery (PET)	211	140	1g mPSL pulse PSL 50 mg TCZ 162 mg	2 weeks	2 years	Discharge (Remission with PSL 3 mg & TCZ)

M, Male; F, Female; GCA, giant cell arteritis; HTN, hypertension; CRP, C-reactive protein; ESR, erythrocyte sedimentation rate; PET, positron emission tomography; PSL, prednisolone; mPSL, methylprednisolone; TCZ, tocilizumab; LV-GCA, large-vessel GCA, C-GCA, cranial GCA; Vx, vaccination; Dx, diagnosis.

## Data Availability

The data presented in this study are available on request from the corresponding author. The data is not publicly available due to privacy restrictions.
